# Comparative ribosome profiling reveals extensive translational complexity in different *Trypanosoma brucei* life cycle stages

**DOI:** 10.1093/nar/gkt1386

**Published:** 2014-01-17

**Authors:** Juan-José Vasquez, Chung-Chau Hon, Jens T. Vanselow, Andreas Schlosser, T. Nicolai Siegel

**Affiliations:** ^1^Research Center for Infectious Diseases, University of Wuerzburg, Wuerzburg 97080, Germany, ^2^Département Biologie cellulaire et infection, Institut Pasteur, Unité Biologie Cellulaire du Parasitisme, Paris 75015, France, ^3^INSERM U786, Paris 75015, France and ^4^Rudolf Virchow Center, University of Wuerzburg, Wuerzburg 97080, Germany

## Abstract

While gene expression is a fundamental and tightly controlled cellular process that is regulated at multiple steps, the exact contribution of each step remains unknown in any organism. The absence of transcription initiation regulation for RNA polymerase II in the protozoan parasite *Trypanosoma brucei* greatly simplifies the task of elucidating the contribution of translation to global gene expression. Therefore, we have sequenced ribosome-protected mRNA fragments in *T. brucei*, permitting the genome-wide analysis of RNA translation and translational efficiency. We find that the latter varies greatly between life cycle stages of the parasite and ∼100-fold between genes, thus contributing to gene expression to a similar extent as RNA stability. The ability to map ribosome positions at sub-codon resolution revealed extensive translation from upstream open reading frames located within 5′ UTRs and enabled the identification of hundreds of previously un-annotated putative coding sequences (CDSs). Evaluation of existing proteomics and genome-wide RNAi data confirmed the translation of previously un-annotated CDSs and suggested an important role for >200 of those CDSs in parasite survival, especially in the form that is infective to mammals. Overall our data show that translational control plays a prevalent and important role in different parasite life cycle stages of *T. brucei*.

## INTRODUCTION

Eukaryotic gene expression is regulated at multiple levels, including extensive and elaborate post-transcriptional regulation that affects RNA stability, protein translation and protein turnover (1). How much these individual levels contribute to the final outcome, the steady state level of a protein, is not known for any organism. The need for regulation beyond the level of transcription and RNA stability, for example at the level of translation or protein stability, has become evident from transcriptome and proteome studies that have revealed only weak correlations between mRNA and protein levels in various organisms, including yeast, humans and the protozoan parasite *Leishmania major* (2–6). Furthermore, mRNA translation has recently been found to be the most extensively regulated step in mammals (7).

*L. major* and the related kinetoplastids *Trypanosoma cruzi* and *Trypanosoma brucei* are the causative agents of leishmaniasis, Chagas disease and sleeping sickness, which are significant human diseases leading to the death of tens of thousands of people worldwide every year (8,9). Interestingly, the genome organization of kinetoplastids differs from that of other eukaryotes in that most protein-coding genes are transcribed from long polycistronic transcription units (PTUs). These PTUs can encompass >100 mostly functionally unrelated genes (10–13). Maturation of polycistronic RNA precursors into functional mRNA transcripts involves *trans*-splicing of a 39 nt leader sequence to the 5′ end and polyadenylation of the 3′ end (14–16). The absence of promoter sequence motifs, the organization of genes in PTUs and an apparently conserved open chromatin structure surrounding RNA polymerase II (pol II) transcription start sites (12,17) strongly indicate a lack of regulation of gene expression at the level of pol II transcription initiation. This lack of transcriptional regulation should greatly facilitate the effort to quantify the extent to which different post-transcriptional mechanisms contribute to gene expression.

Despite the lack of transcriptional control, *L. major*, *T. cruzi* and *T. brucei* are capable of tightly regulating gene expression throughout their complex life cycles, which requires the adaptation of the parasites to the large environmental differences found between the insect vectors and mammalian hosts (18,19). In *T. brucei*, these differences include, among others, a change in temperature from 37°C in the mammalian host to 27°C in the insect vector and a change in the availability of glucose, the preferred energy source of the parasite (20,21). Consequently, significant effort has been invested in elucidating the post-transcriptional mechanisms of gene control in these parasites. Work from several laboratories has led to the identification of sequence motifs capable of modulating RNA stability or translational efficiency in a life cycle-specific manner (22–27), a bias in codon usage suggested to affect translational efficiency (28), and a genome-wide analysis of mRNA stability has found half-lives among transcripts to range over two orders of magnitude in *T. brucei* (29).

A comparable study to evaluate the degree of regulation at the level of protein translation has not been performed even though multiple observations suggest translational control as an important regulator of gene expression. For example, kinetoplastid genomes encode more proteins involved in translation initiation control than those of yeast or many metazoans, including two to three homologues for poly(A) binding proteins as well as four isoforms of the translation initiation factor (eIF4E) and five eIF4G isoforms (30–34). Moreover, RNA-sequencing analyses performed in *T. brucei* revealed widespread alternative *trans*-splicing, resulting in multiple transcripts, with varying 5′ untranslated region (UTR) lengths for a particular gene (13,35,36). These findings suggest that variations in 5′ UTR lengths may lead to the in- or exclusion of regulatory elements that influence translational efficiency (37). Such regulatory elements could be small, so-called upstream open reading frames (uORFs) or micro ORFs, which are ORFs located in the 5′ UTR of mRNA. In eukaryotes, translation initiation involves the recruitment of a pre-initiation complex to the 5′ end of mRNA, followed by scanning of the UTR to locate the first AUG start codon (38). uORFs can affect the scanning process and have been shown to influence translation of the downstream coding sequence (CDS) by affecting both the rate and the site of translation initiation (39,40). Based on RNA-sequencing data, in *T. brucei* ∼20% of 5′ UTRs contain at least one uORF (35,36). In addition, it has been shown for a luciferase reporter that the removal of an upstream start-codon can lead to a 7-fold increase in protein levels (41).

In the past, approaches to identify and quantify translated RNA transcripts have focused on the isolation of RNA transcripts from polyribosome fractions (42). While this approach has been successfully used to demonstrate differential translation of RNA in *T. brucei* (43,44), we have adapted a higher resolution approach termed ribosome profiling that was recently developed (45). This approach is based on high-throughput sequencing of ribosome-protected RNA fragments, so-called ribosome ‘footprints’. RNA not protected by ribosomes is removed by nuclease digestion; ribosome footprints are converted into libraries of DNA molecules suitable for high-throughput sequencing, and the abundance of individual footprints is determined by deep sequencing. In yeast, mice and *Escherichia coli*, measurements of the average ribosome occupancy (ribosome density profiles) have been successfully used to estimate the rates of translation (rates of protein synthesis) (45–47). Ribosome density, while not perfect, has been shown to be a much better predictor of protein levels than measurements of mRNA levels (45).

Importantly, this approach not only provides quantitative information on the number of RNA molecules associated with ribosomes, it also yields position-specific information regarding the location of ribosomes on mRNA transcripts. This information is critical, as association of an RNA transcript with a ribosome does not necessarily mean that the transcript is being translated. The ribosome could be associated with the 5′ UTR or it could be stalled, failing to produce functional protein. In addition, ribosome positions can be used to accurately map the correct CDSs and help identify novel CDSs that have been missed in previous genome annotations (48).

Here we report the adaptation of a ribosome profiling approach to *T. brucei*. Comparative ribosome profiling analyses of the bloodstream and procyclic parasite forms allowed us to generate a genome-wide picture of translation. We observed a 100-fold range in translational efficiency among genes and life cycle-specific differences in translational efficiency for a large number of genes. In addition, our ribosome profiling data enabled the identification of hundreds of previously un-annotated CDSs and incorrectly annotated translation initiation sites, and suggest a regulatory role of uORFs.

## MATERIALS AND METHODS

### *T. brucei* culture and cell harvest

The procyclic form (PF) of *T. brucei* strain Lister 427 were cultured at 27°C in SDM-79 supplemented with 10% fetal bovine serum and hemin (7.5 mg/l) medium (21) up to a density of 10^7^ cells/ml. Wild-type bloodstream form (BF) of Lister 427 (MITat 1.2 clone 221) were cultured at 37°C in HMI-11 up to a cell density of 1.5 × 10^6^ cells/ml. Two minutes before harvest, cycloheximide was added to a final concentration of 100 µg/ml. Cells were collected by centrifugation at 3000 × g, 4°C for 5 min, washed with polysome lysis buffer (10 mM Tris-HCl pH 7.4, 300 mM KCl and 10 mM MgCl_2_), transferred to a 1.5-ml microcentrifuge tube and pelleted. To lyse the cells, 360 µl polysome lysis buffer, 40 µl of 10% n-octylglycoside and 20 units of TURBO DNaseI (Ambion) were added per 10^9^ cells and incubated for 30 min on ice. The lysate was centrifuged at 16 000× g, 4°C for 10 min, the supernatant transferred to a new microcentrifuge tube and the OD_260_ was determined using a Nanodrop 2000.

### Preparation of RNA footprint and mRNA sequencing libraries

For both BF and PF, 200 µl aliquots of the lysate (OD_260_ = 40) were digested with RNase I (Ambion) at RT (1200 units) or on ice (1600 units). After 1 h, the digestions were stopped by adding 100 units of SUPERase•In RNase inhibitor (Ambion) to the RNase-treated samples. In parallel, 100 units of SUPERase•In RNase inhibitor were added to a 200 µl aliquot of lysate not containing RNase I (undigested control). Monosomes were purified using sucrose gradients as described previously (45).

Total RNA from undigested lysates and the footprints collected in the monosome fractions were purified using hot acid Phenol–Chloroform–Isoamyl alcohol (v/v/v 25:24:1) at 65°C (49). To generate mRNA sequencing libraries, undigested RNA was polyA-enriched using a Dynabeads® mRNA Purification Kit (Ambion) according the manufacturer’s instructions. The polyA-enriched RNA was fragmented by incubation with an RNA Fragmentation Reagent (Ambion) at 70°C for 30 min. Successful fragmentation was monitored on a 15% denaturing-PAGE gel. Both, ribosome footprints and fragmented mRNA (26–34 nt) were size-selected by electrophoresis using a 15% denaturing-PAGE gel and two custom-made (IDT) synthetic RNA markers [5′-AUGUACACGGAGUCGAGCUCAACCCGCAACGCGA-(Phos)-3′] and [5′-AUGUACACGGAGUCGACCCAACGCGA-(Phos)-3′].

Sequencing libraries were prepared as described (50) except for the omission of an rRNA depletion step during the footprint library preparation and the use of KAPA HiFi polymerase (Kapa Biosystems) for the final amplification step.

### Pre-processing and mapping of read

Reads from all libraries were processed and mapped using the same parameters. Adapter sequence (i.e. CTGTAGGCACCATCAAT) was trimmed from the 3′ end of reads using cutadapt (http://code.google.com/p/cutadapt/) and reads shorter than 20 nt after trimming were removed. Trimmed reads were mapped to the reference genome using bowtie-2 with default ‘local-sensitive’ mode (51). Genome, as well as gene annotations, of strain 927 version 4.2 were downloaded from EuPathDB (52) and used as the reference in all analyses.

### Calculation of abundance, translational efficiency and read 5′-end periodicity

A read is considered to map to a region when its midpoint falls within the annotated range. Abundance of a region in a library was defined as reads mapped per kilobase per million non-structural RNA reads (rpkm). Non-structural RNA refers to the reads mapped to the genome excluding annotated rRNA and tRNA regions. Translational efficiency of a region was defined as its ratio of abundance in the ribosome profiling library to that of the control RNA library of the same life cycle stage. To investigate the distinct 3-nt periodicity of the ribosome profiling libraries, 5′ ends of the mapped reads were piled up and the piled-up coverage of each position from each gene (using either start or stop codon as the reference point) were summed up in meta-gene analyses. Genes with less than 10 reads within the plotted region were excluded.

### Definition of upstream ORFs and novel CDSs

A uORF was defined as any ORF ≥9 nt that was located within an annotated 5′ UTR and was on the same strand as the annotated ORF. Novel CDSs are defined as any translated ORF ≥ 30 nt located at least 20 nt away from any existing annotations. In cases in which multiple overlapping novel ORFs were found on different frames of the same strand, only the longest were retained. An ORF is defined as translated when ≥ 70% of its CDS is being covered by the pooled ribosome profiling reads (i.e. four libraries) with ≥2 reads per nucleotide.

### Calculation of ribosome release score

Ribosome release scores (RRSs) of all ORFs, including annotated CDSs, putative CDSs and uORFs, were calculated as described with slight modifications (53). Briefly, a pseudo 3′ UTR region (pUTR3) was first assigned to all ORFs. pUTR3 of an ORF was defined as the region between its stop codon and a downstream start codon in any of the three frames on the same strand. Reads mapped within the CDSs and pUTR3 per kilobase (i.e. CDS_rpk_ and pUTR3_rpk_) in both ribosome profiling and RNA-sequencing control libraries were calculated for each ORF. The ratio of CDS_rpk_ to pUTR3_rpk_ in both ribosome profiling and RNA-sequencing control libraries (i.e. ribo_ratio_ and RNA_ratio_) were calculated. RRS of each ORF was then defined as the ratio of ribo_ratio_ to RNA_ratio._

### Identification of novel putative CDSs important for parasite’s fitness

We reanalysed the data of a published genome-wide RNA interference fitness–costs association study (54) with our newly defined CDSs. Briefly, short read data were downloaded from European Nucleotide Archive under accession number ERP000431. Reads were then mapped to the genome using bowtie2 with default ‘local-sensitive’ mode. The number of reads mapping to the annotated and putative CDSs was counted. To identify those putative CDSs that have significantly less reads mapped in the RNA interference libraries, which potentially contribute to the parasite’s fitness, we performed differential read count analyses on the pair-wise library comparisons (listed below) using three softwares, including DESeq1 (55), DESeq2 (http://www.bioconductor.org/packages/release/bioc/html/DESeq2.html) and EdgeR (56), with default settings. CDSs that were identified to have significantly less reads in the RNA interference libraries by all three algorithms (*P* < 0.05 in each case) linear fold change ≥ 5 were considered to be potentially related to the loss of fitness. The pair-wise library comparisons include BF uninduced versus BF induced for 6 days, BF uninduced versus differentiated cells induced and BF uninduced versus PF induced.

### Analysis of proteomics data

All mass spectrometric raw data files from Butter *et al.* (57) were processed as described except that the data were searched additionally with the previously un-annotated CDSs listed in Supplementary Tables S4 and S5. Briefly, MaxQuant 1.4.1.2 including the Andromeda search engine (58,59) was used for processing raw data and database searching. The experimental design template used was as described before (57). The search was performed against a combination of three databases: a *T. brucei* database containing 19 103 annotated protein entries for strains 427 and 927 (version 5.0, Tb427 and Tb927, downloaded from http://tritrypdb.org), a list of uORFs and putative CDSs identified in this study (Supplementary Tables S4 and S5) and a database containing typical contaminants. Enzyme search specificity was Trypsin/P with three miscleavages for tryptic digests and LysC with two miscleavages for digests with lysyl endopeptidase. Carbamidomethylation on cysteines was set as fixed modification; methionine oxidation and protein N-terminal acetylation were considered as variable modifications. Mass accuracy tolerances (after recalibration) were 6 ppm for precursor ions, and 0.5 Da for CID MS/MS and 20 ppm for HCD MS/MS spectra, respectively. False discovery rate was fixed at 1% at peptide and protein level, with posterior error probability (PEP) as sorting criterion. Identifications were matched between runs within a 2-min window. Protein groups identified in the potential CDS database had at least one unique peptide and a PEP < 0.0005.

## RESULTS

### Ribosome footprints reveal ribosome position at sub-codon resolution

To obtain genome-wide information on protein synthesis, we adapted a ribosome profiling protocol published in yeast and mice (45,46) for use in *T. brucei*. We sequenced ribosome footprints and fragmented mRNA from the two trypanosome stages that have been adapted to *in vitro* culture, the PF of the tsetse fly midgut and the proliferating BF that lives in the blood of the mammalian host. Cultured cells were treated with cycloheximide to arrest translating ribosomes and harvested. Because cycloheximide has been shown to stabilize RNA transcripts in *T. brucei* (60), the treatment was limited to 2 min and applied to both, cells used for ribosome profililng and cells used for RNA-sequencing analyses. To maximize the accuracy of the ribosome footprints, we optimized the digestion efficiency by testing different nuclease concentrations and reaction temperatures (‘Materials and Methods’ section and Supplementary Figure S1). Next, to map translated regions of RNA and to exclude RNase-protected regions that are scanned by the 43S pre-initiation complex, we used sucrose gradients to specifically enrich for monosomes (Supplementary Figure S1). The high reproducibility between libraries prepared under different conditions demonstrated the robustness of the ribosome profiling approach despite the elaborate library preparation protocol (Supplementary Figure S2, BF digestion at 4°C versus digestion at room temperature, R^2^ = 0.9755).

**Figure 1.**
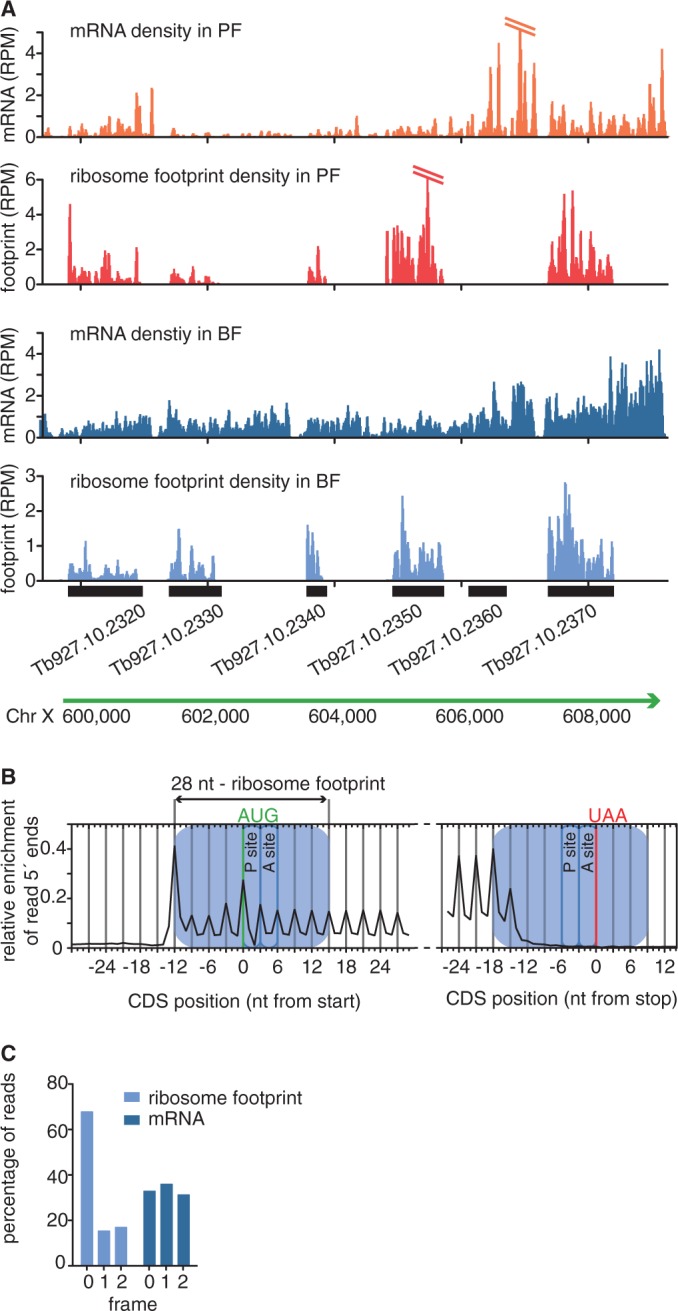
Ribosome footprints reveal coding sequences at sub-codon resolution. (**A**) Ribosome footprints are strongly enriched across CDSs. mRNA densities and ribosome densities are shown as reads per nucleotide per million reads (RPM) to normalize for differences in library size. (**B**) Alignment of the 5′ nucleotide from ribosome footprint reads that map close to translation start or translation termination sites. Blue boxes mark the approximate size of the ribosome footprint. (**C**) Percentage of position of sequence reads relative to reading frame.

To ensure that nuclease-resistant RNA fragments represent ribosome footprints and not short RNA fragments protected by other RNA-binding proteins, we compared the genome-wide distribution of sequence reads obtained from ribosome profiling and RNA-sequencing libraries. Alignment of ribosome profiling reads revealed that the nuclease-protected reads predominantly mapped along CDSs with very few reads aligning to intergenic regions (Figure 1A). The 5′ nucleotide of the ribosome profiling reads aligned from 12 nt upstream of the ATG codon to ∼18 nt upstream of the stop codon (Figure 1B). Given a footprint length of 28–30 nt, the ribosome protects mRNA from 12 nt upstream of the start codon to 9–11 nt downstream of the stop codon. In contrast to ribosome footprint reads, RNA-sequencing reads were aligned along the CDSs and UTRs, as expected (Figure 1A). In addition to the enrichment across CDSs, the alignment of ribosome profiling reads revealed a distinct 3-nt periodicity, with 68% of BF ribosome profiling reads starting at the first nucleotide of a codon (Figure 1B and C and Supplementary Figure S3). No such 3-nt periodicity was observed for the mRNA sequence reads.

**Figure 2.**
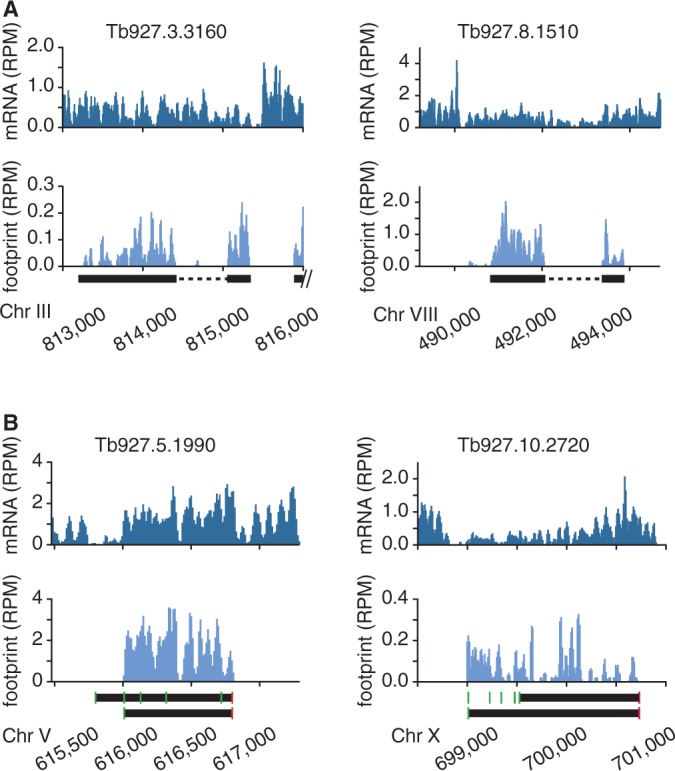
Ribosome footprints reveal translated regions. (**A**) Ribosome profiles for the two intron-containing genes. Introns are represented as a dashed line. (**B**) Ribosome profiles of two possibly mis-annotated CDSs. Black bars mark annotated CDSs. Grey bars mark CDSs predicted based on ribosome profiles. Green boxes represent ATG codons and red boxes represent stop codons.

Only two *T. brucei* genes have introns, one encoding a poly(A) polymerase and the other encoding a DNA/RNA helicase (61,62). Surprisingly, there is almost no decrease in sequence reads detectable by RNA-sequencing across the introns, while the ribosome profiling data clearly show that the intronic RNA is not translated (Figure 2A). These data indicate that the process of *cis*-splicing must be unusually inefficient in trypanosomes, but that a tight control mechanism ensures that only mature mRNAs enter the translational machinery.

The strong enrichment of ribosome profiling reads across CDSs, the lack of ribosome profiling reads across introns and the observed 3-nt periodicity are characteristic of ribosome-protected RNA footprints and argue against unspecific RNA protection (45). Thus, ribosome profiling reads indeed represent ribosome footprints and provide a means to measure protein synthesis and, in addition, to identify both previously unannotated CDSs as well as non-translated transcripts previously annotated as coding (for example Figure 1A, Tb927.10.2360). The latter may be either incorrectly annotated or not translated in the two life cycle stages examined.

### Identification of translation initiation sites

Genome-wide mapping of spliced leader acceptor sites (SASs), i.e. the 5′ end of the 5′ UTR to which the spliced leader RNA is *trans*-spliced, revealed hundreds of instances in which SASs mapped within annotated CDSs or far upstream of annotated CDSs (13,35,36). These findings indicated that for many genes the true CDS was shorter than the annotated CDS while, for others, it allowed the possibility of translation initiation at an AUG upstream of the annotated translation initiation site. However, while RNA-sequencing allowed the unambiguous identification of mis-annotation for many genes, translation initiation does not always occur at the first AUG downstream of the SAS. Thus, the usefulness of RNA-sequencing to identify the true translation initiation sites is limited and depends on the genomic context.

In contrast to RNA-sequencing, ribosome profiling allows the determination of the translational landscape. Thus, it allows the identification of CDS mis-annotations and the determination of the most probable translation initiation sites. Exemplary for numerous genes, for Tb927.5.1990 we detected no translation between the first and the second AUG (green bars) indicating that this region is not translated in the BF and PF and that translation initiates at the second AUG (Figure 2B, left panel). For Tb927.10.2720 we observed strong translation upstream of the annotated CDS. The fact that translation starts at an upstream AUG, which is in frame with the annotated translation initiation site, suggests that the CDS of Tb927.10.2720 is longer than annotated (Figure 2B, right panel). These examples show that ribosome profiling data can be used for the re-annotation of incorrectly annotated CDSs.

### Ribosome profiling reveals extensive translational control

While the role of RNA stability has been investigated on a genome-wide scale in *T. brucei* (29), global regulation at the levels of protein translation has not been analysed. Therefore, we decided to apply the ribosome profiling approach to evaluate the degree of regulation occurring at the level of translation in trypanosomes.

To estimate the rate of protein synthesis in trypanosomes, we determined the ribosome footprint density across CDSs [measured as reads per million reads per kb (rpkm), see ‘Materials and Methods’ section]. Of 77 million PF and 44 million BF ribosome footprint sequence reads, 28% (PF) and 14% (BF) could be aligned to annotated CDSs, while 70% (PF) and 85% (BF) mapped to known structural non-coding RNA (ncRNA). The high percentage of structural ncRNA in the footprint fraction has been reported previously (45) and results from the large amount of rRNA in the monosome fraction. The lower percentage of CDS reads in the BF sample compared with the PF sample may be due to libraries being generated from the wild-type 427 strain while reads were aligned to the better-annotated genome of the TREU 927 strain. In the BF, some of the most highly expressed genes encoding for variant surface glycoproteins differ in sequence between the 427 and 927 strains, and footprints from these genes were not aligned in this analysis.

**Figure 3.**
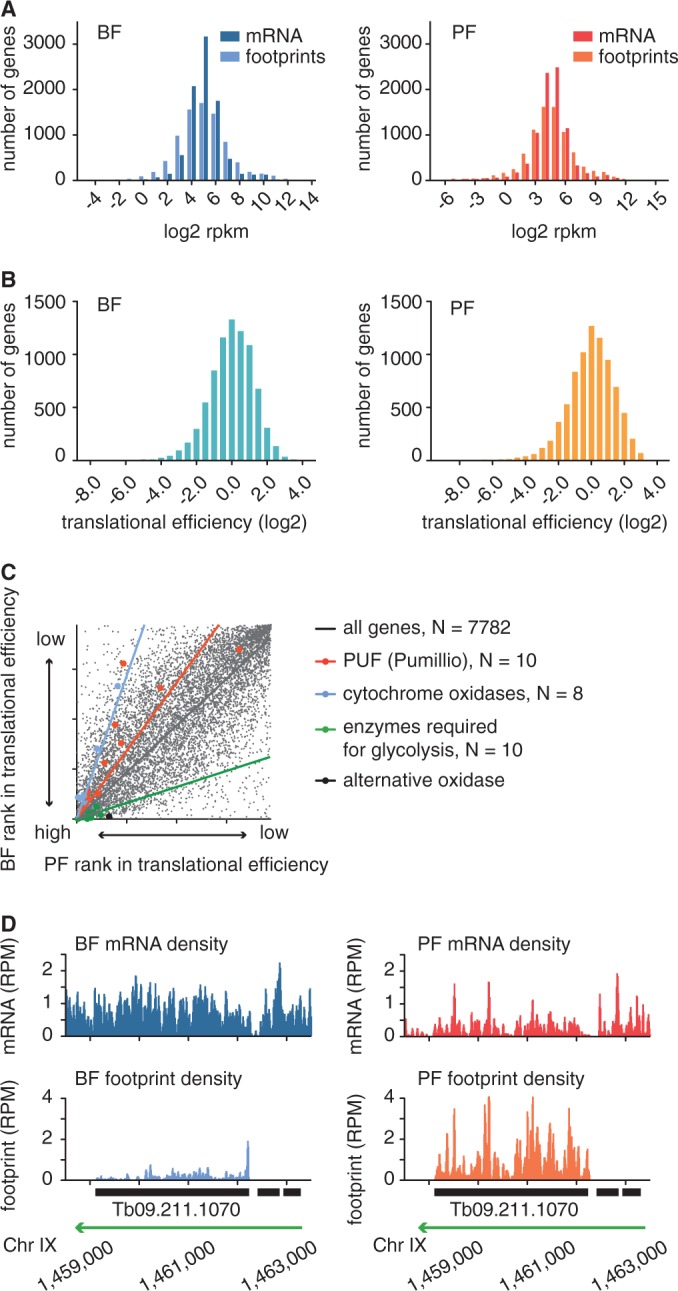
Translational efficiency is regulated. (**A**) Histograms of mRNA abundance and ribosome density (rate of protein synthesis) for BF (left panel) and PF parasites (right panel). (**B**) Histogram of translational efficiency (ratio of ribosome footprint density to mRNA abundance) for BF (left panel) and PF parasites (right panel). (**C**) Pair-wise comparisons of translational efficiency in PF and BF. CDSs were ranked based on translational efficiency (1 = highest translational efficiency, 7782 = lowest translational efficiency) in BF and PF. Ranks between life cycle stages show a correlation of R = 0.7428. Gene families with developmentally regulated translational efficiencies are colour coded: Pumillio genes (red), cytochromes oxidase (blue) genes required for glycolysis (63) (green) and the alternative oxidase (Tb927.10.7090, black). (**D**) Ribosome footprint profile of a gene with developmentally regulated translational efficiency. Green arrow indicates direction of transcription.

Nevertheless, for 8072 genes (82% of annotated CDSs), we were able to detect ≥ 10 ribosome footprint reads, a threshold that we defined as translation (Supplementary Table S1). We found the rate of translation to differ greatly among proteins. In the PF, we observed a 11 448-fold difference in ribosome density (3548-fold difference in mRNA levels) between the 1% most highly and the 1% most weakly translated proteins, while in the BF we observed a 1623-fold difference in ribosome density and a 262-fold difference in mRNA levels (Figure 3A). These findings suggest translational efficiency to significantly contribute to the regulation of gene expression.

To determine the translational efficiency for individual genes, we normalized for differences in mRNA abundance, i.e. we divided the ribosome footprint density by the levels of mRNA abundance. Our ribosome profiling data indicate a relatively unbiased distribution of ribosome density across CDSs in the BF and a very minor increase in density towards the 5′ end of CDSs in the PF (Supplementary Figure S3). Nevertheless, whenever determining the translational efficiency we excluded mRNA and footprint reads that mapped to the first 40 nt of a CDS to avoid possible artifacts from ribosome stalling at translation initiation sites. Between the 1% most efficiently and the 1% least efficiently translated proteins, we observed in the PF a 117-fold and in the BF a 64-fold range in translational efficiency, i.e. in the amount of proteins produced per transcript (Figure 3B and Supplementary Table S2). This range in translational efficiency is similar to what has been observed in yeast and roughly 10-fold higher than what has been described for mice (45,46). Interestingly, we observed no correlation between RNA abundance and translational efficiency (R^2^ = 0.03), suggesting that translational efficiency is regulated independently of RNA stability.

### Life cycle-specific regulation of translational efficiency

Next we addressed the question if translational efficiency is regulated in a life cycle-specific manner. Ribosome density measurements only allow meaningful comparisons of the rate of protein synthesis when the speed of translation is assumed to remain constant. This assumption is supported by measurements in mouse embryonic stem cells (46,64); however, the speed of translation may very well not be identical in two life cycle stages that live at different temperatures. In addition, our current protocol does not allow the absolute quantification of mRNA and ribosome footprint levels in the two different life cycle stages. Therefore, we decided to compare the overall changes in translational efficiency within each life cycle stage. To this end we ranked the genes in each life cycle stage based on their relative translational efficiency and compared their rank between the two life cycle stages. A pair-wise comparison indicated a generally positive correlation between translational efficiency in the two stages (Figure 3C, Pearson’s correlation coefficient = 0.7428, *P* < 0.0001, 95% CI 0.7327–0.7526), but it also revealed distinct life cycle-specific differences for subsets of genes. For example, 58 genes found in the lowest 25% in terms of translational efficiency in the PF were among the top 25% most efficiently translated proteins in the BF. These contain various RNA binding proteins, numerous phosphatases and expression site-associated genes (Table 1 and Supplementary Table S3). For an example of a gene more efficiently translated in the BF than in the PF see Figure 3D.

**Table 1.** Developmentally regulated translation

Gene ID	Description	BF rank	PF rank	Change in rank (PF–BF)
Translation up-regulated in BF				
Tb927.7.3250	Expression site-associated gene 6 (ESAG6) protein, putative	1372	7405	6033
Tb927.4.3980	Chaperone protein DNAj, putative	1301	7248	5947
Tb927.10.4780	GPI inositol deacylase precursor (GPIdeAc)	390	5994	5604
Tb927.8.8140	Small GTP-binding rab protein, putative	1217	6804	5587
Tb927.6.3480	RNA-binding protein, putative (DRBD5)	1856	7262	5406
Tb11.01.4701	Membrane-bound acid phosphatase 1 precursor (MBAP1)	689	6065	5376
Tb927.1.4650	Cyclin-like F-box protein (CFB2)	10	5289	5279
Tb927.3.5660	UDP-Gal or UDP-GlcNAc-dependent glycosyltransferase, putative	797	6041	5244
Tb927.2.6000	Glycosylphosphatidylinositol-specific phospholipase C (GPI-PLC)	1634	6793	5159
Tb927.4.5310	Serine/threonine-protein kinase a, putative	1656	6776	5120
Translation up-regulated in PF				
Tb927.5.440	trans-Sialidase, putative	6785	418	−6367
Tb927.7.6850	trans-Sialidase (TbTS)	7513	1445	−6068
Tb927.7.7470	Receptor-type adenylate cyclase GRESAG 4, putative	7001	1268	−5733
Tb927.1.2120	Calpain, putative	6258	739	−5519
Tb927.4.360	1,2-Dihydroxy-3-keto-5-methylthiopentene dioxygenase, putative	6587	1089	−5498
Tb09.160.5550	Calpain-like cysteine peptidase, putative	6936	1868	−5068
Tb927.8.7690	Amino acid transporter (pseudogene), putative	6693	1731	−4962
Tb927.8.1610	MSP-B, putative	7199	2272	−4927
Tb11.01.6650	Serine/threonine-protein kinase, putative	5280	363	−4917
Tb927.7.7110	Leucine-rich repeat protein (LRRP), putative	6501	1601	−4900
Genes for which translational up-regulation in PF was previously shown				
Tb927.10.280	Cytochrome oxidase subunit VI (COXVI)	2820	810	−2010
Tb09.160.1820	Cytochrome oxidase subunit V (COXV)	935	291	−644
Tb927.10.14000	Aconitase (ACO)	657	31	−626
Tb927.6.510	GPEET2 procyclin precursor	3848	365	−3483
Tb927.10.10260	EP1 procyclin (EP1)	4682	34	−4648
Tb927.10.10220	Procyclin-associated gene 2 (PAG2) protein (PAG2)	6903	5375	−1528
Tb927.5.330	Receptor-type adenylate cyclase GRESAG 4, putative	3779	809	−2970

List of genes with highest change in translational efficiency between PF and BF. List does not include hypothetical genes.

Based on mRNA and protein level measurements, differential translational efficiency has been predicted for aconitase, the cytochrome oxidases V and VI and procyclins (22,25,65). For all of these genes, our data indicate an increased translational efficiency in the PF (Table 1), thus confirming previous observations and validating our measurements. For selected groups of proteins, e.g. annotated as cytochrome oxidase or those required for glycolysis, we noticed the translational efficiency to correspond to the changes that have been described for the glucose metabolism (Figure 3C). Whereas the BF is entirely dependent on the glycolytic pathway to generate energy, the PF living in the insect midgut, where glucose availability is uncommon or absent, has been shown to contain a much more elaborate energy metabolism (66). In contrast, the efficient translation of the group of PUF (Pumilio and FBF) proteins in PF was unexpected (Figure 3C). PUF proteins regulate translation and mRNA stability by binding sequences in their target RNAs and have been shown to affect gene expression in organisms as divergent as *Plasmodium falciparum*, *T. brucei*, humans and yeast (67–69). Interestingly, we found both poly(A) binding proteins, all four isoforms of eIF4Es and four of the five eIF4G isoforms, all proteins involved in control of translation initiation, to be more efficiently translated in the PF than in the BF (Supplementary Table S2).

Given the large dynamic range and the life cycle-specific regulation, we propose that differences in translational efficiency contribute substantially to the control of gene expression in *T. brucei*.

### uORFs are frequently translated and may regulate translational efficiency

The broad ∼100-fold range in translational efficiency among genes and the differences in translational efficiencies between life cycle stages raise the question of how protein translation is regulated. For a small number of genes in *T. brucei*, sequence motifs in the 3′ UTR have been linked to developmentally regulated changes in translational efficiency, but for the large majority of genes no such motifs have been identified (70). Numerous reports from other eukaryotes demonstrated that uORFs can function as important regulators of gene expression (71). For example, analysis of mRNA and protein levels from >10 000 mammalian genes revealed that the presence of an uORF correlates with a significant reduction of expression from the downstream CDS. Furthermore, using reporter constructs, it was estimated that the presence of an uORF results in a decrease in gene expression of 30–80% with only a minor reduction of mRNA levels (72). No such analyses have been performed for *T. brucei*, but it has been shown that the removal of a uATG leads to a 7-fold increase in protein levels of a luciferase reporter (41). In this manuscript, we use the term ORF or uORF to refer to regions of DNA sequences beginning with an ATG and ending with a termination codon that may or may not encode for proteins. The term coding sequence (CDS) is used to refer to ORFs that encode proteins. Minimally, uORFs consist of 9 nt, containing an upstream start codon (uATG), an additional sense codon and a termination codon, whereby the termination codon may be located downstream of the start codon of the main CDS (73).

Even though 5′ UTRs have only been assigned for 4909 genes (927 version 4.2), applying the criteria listed above, we identified 8310 uORFs and found 1092 (22%) of 5′ UTRs to contain at least one uORF (Supplementary Table S4). Thus, uORF are much less common in *T. brucei* than in mammals where 40–50% of genes contain uORFs (74,75). Nevertheless, while uORFs cannot explain the translational regulation for all transcripts, they may represent one of several factors important for the regulation of gene expression in *T. brucei*.

**Figure 4.**
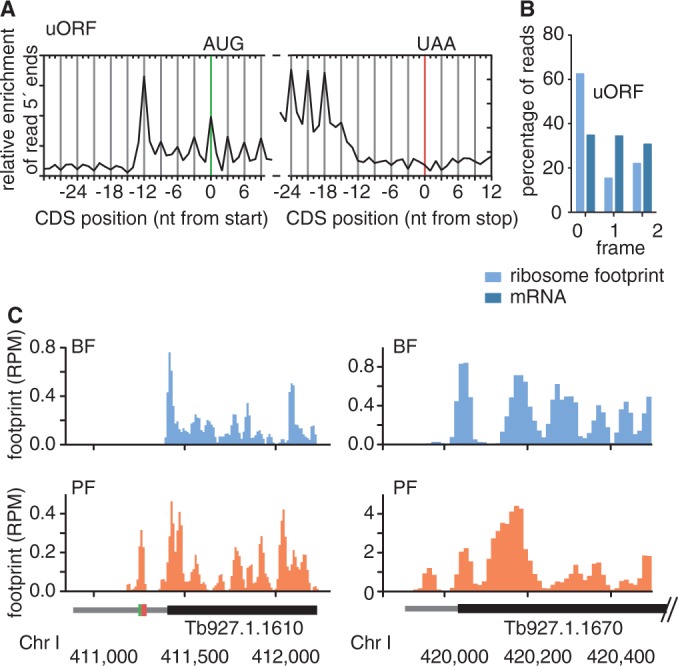
Ribosome footprints reveal translation of uORFs. (**A**) Alignment of the 5′ nucleotides from ribosome footprint reads that map close to translation start or translation termination sites of uORFs. (**B**) Percentage of position of sequence reads relative to reading frame. (**C**) Ribosome profiles of two genes with uORF (left panel) and without uORF (right panel). Narrow grey boxes represent 5′ UTRs, green box represents AUG-codon, red box represents termination codon.

To determine the degree to what uORFs are translated, a prerequisite for affecting translational efficiency of the downstream CDS, we calculated the ribosome density across uORFs and identified 1834 uORFs (22%) with ≥ 2× read-coverage and ≥70% of the ORF covered. The average length of these uORFs was 22 aa. Just like for annotated CDSs, the alignment of ribosome profiling reads across these uORFs revealed a distinct 3-nt periodicity, with 63% of BF ribosome profiling reads starting at the first nucleotide of a codon (Figure 4A and B). While the periodicity of ribosome footprints across uORFs was slightly less pronounced than for annotated CDS, our data suggest that a large proportion of uORFs is translated.

To evaluate the regulatory potential of uORFs, we compared the ribosome densities between transcripts containing at least one uORF (*N* = 1092) and those without uORFs (*N* = 3817). In the BF, median ribosome density across the CDSs of transcripts with uORFs was 30.31 rpkm compared with 44.86 rpkm for genes without uORFs (Table 2). In the PF, median ribosome densities were 20.01 rpkm (genes with uORF) and 36.50 rpkm (genes without uORF). Thus, in both life cycle stages we observed a higher ribosome density for genes without uORFs than for genes with uORFs (*P* < 0.0001). While we also observed a higher mRNA level for genes without uORFs compared with genes with uORFs (*P* < 0.0001), the differences in mRNA levels were lower than the differences in ribosome density (Table 2). Thus, our data indicate that median translational efficiency is higher for genes without uORF than for genes with uORF, suggesting that the presence of uORFs may negatively impact translation of downstream CDSs.

**Table 2.** Translational efficiency of genes with and without uORF

	Transcripts with uORF (*N* = 3817)	Transcripts without uORF (*N* = 1092)	Mann–Whitney test
Bloodstream form			
Ribosome density (median rpkm)	30.31	44.66	*P* < 0.0001
mRNA levels (median rpkm)	30.83	36.13	*P* < 0.0001
Translational efficiency (ribosome density/mRNA levels)	1.00	1.27	*P* < 0.0001
Procyclic form			
Ribosome density (median rpkm)	20.01	36.50	*P* < 0.0001
mRNA levels (median rpkm)	24.04	30.38	*P* < 0.0001
Translational efficiency (ribosome density/mRNA levels)	0.87	1.26	*P* < 0.0001

Reads mapping within the first 40 nt of a CDS were not considered for the measurements of translational efficiency. Number of genes with annotated 5′ UTR, *N* = 4909.

Noteworthy, while median ribosome density for CDSs was higher in the BF than in the PF (Table 2), we observed the opposite for 5′ UTRs (*P* < 0.0001). For 5′ UTRs we found a higher ribosome density in the PF (median rpkm: 7.73) than in the BF (median rpkm: 2.89, Supplementary Table S4, for an example see Figure 4C). In addition, we found that in the BF 5′ UTRs with uORF (4.08 rpkm) have a higher ribosome density than 5′ UTRs without uORFs (2.54 rpkm; *P* < 0.0001). Unexpectedly, the contrary was true in PF. In the PF we found 5′ UTRs without uORF (8.34 rpkm) to have a higher ribosome density than 5′ UTRs with uORF (5.01 rpkm; *P* < 0.0001). For an example of a non-AUG uORF see Figure 4C, right panel. Many of such non-AUG uORFs have been observed in yeast and mice embryonic stem cells (mESCs). Interestingly, an increase in 5′ UTR translation, similar to what we observed in the PF compared with the BF, was observed in yeast on starvation and a decrease in 5′ UTR translation was observed on differentiation of pluripotent mESCs into embryoid bodies (45,46).

Genome-wide analyses to prove or disprove a direct correlation between translation of uORFs and translational efficiency is complicated by the existence of multiple 5′ UTR isoforms, resulting from widespread differential *trans*-splicing (35,36,76). At the same time the heterogeneity in UTR lengths raises the intriguing possibility that inclusion or exclusion of an uORF during alternative *trans*-splicing may represent a regulatory mechanism to modulate translational efficiency.

### Ribosome footprints reveal the presence of hundreds of previously un-annotated CDSs

Even though small proteins (<200 aa) have been shown to play major roles in plant and animal development (77,78), the complexity of the short proteome remains largely unexplored because algorithms to reliably predict short CDSs are lacking (79). We observed that while almost all ribosome footprints aligned to annotated features, in the BF 101 197 reads (0.23%) and in the PF 1 116 030 reads (1.44%) did not. It is noteworthy that, just as we observed for 5′ UTRs, the percentage of reads not aligning to CDSs is higher in the PF than in the BF. We suspect that many of these reads originate from un-annotated UTRs but some may stem from previously un-annotated CDSs. A previous RNA-sequencing-based analysis detected 1114 new, un-annotated transcripts in the PF, 1011 of which had the potential to encode one or more peptides (≥ 25 aa). Using available proteomics data, the authors were able to confirm translation for 19 of the 1114 transcripts (13). Given our ability to determine ribosome positions at nucleotide resolution, we looked for new, previously un-annotated CDSs.

We mapped ribosome footprint reads from the BF and PF and determined the ribosome footprint density for all potential CDSs at least 10 aa in length. To avoid overlap with annotated CDSs, we only considered ORFs located at least 20 nt away from annotated features. This approach enabled us to identify a set of 2021 candidate CDSs with ≥2× read-coverage and ≥70% of the ORF covered. The candidate CDSs ranged in length from 10 to 378 aa (average 30 aa, Supplementary Table S5). For 797 of the 1114 previously identified transcripts, we found the average ribosome footprint read-coverage to be ≥2× (Supplementary Table S6. Previously identified transcripts from chromosome 10 were not considered because our sequencing reads were mapped to a newer version of the chromosome 10 assembly and thus genomic coordinates might be incompatible.).

To determine whether our set of candidate CDSs is translated, we analysed published proteomics datasets (57) to search for protein products. For 24 of the 2021 candidate CDSs (average size 117 aa, 13 kDa), protein products could be identified; however, four of these identified peptides also matched annotated CDSs. In addition, we identified protein products for 31 uORFs (Supplementary Table S4). We suspect that the low number of identified protein products relative to the large number of candidate CDSs may be caused, (i) by the experimental set-up of the original proteomics analysis where proteins were separated by 1D-SDS-PAGE prior to in-gel protease digest and mass spectrometric analysis whereby proteins with a size smaller than 5–10 kDa typically run out of the gel, (ii) by the limited sensitivity of mass spectrometric analyses compared with DNA sequencing-based techniques and (iii) by the fact that not all ribosome-protected RNAs are translated.

The first point is supported by the fact that only 67 (3%) of our candidate CDSs were ≥90 aa (≥10 kDa) but for 28% of those large candidate CDSs we could identify peptides. Evidence supporting the possibility that not all ribosome-protected RNAs are translated exists in mice where even known long ncRNAs have been found to be associated with ribosomes (46). To evaluate the coding potential of a transcript and to separate long ncRNA from small coding genes, a new metric was established, the so-called RRS (53). The RRS takes advantage of the fact that translating ribosomes are released on encountering a stop codon. This release results in a sharp decrease in ribosome occupancy between protein-coding regions and the subsequent 3′ UTR. Consequently, the RRS was defined as the ratio of footprint reads in the putative CDS to footprint reads in the corresponding 3′ UTR divided by the ratio of RNA reads in the putative CDS to RNA reads in the corresponding 3′ UTR (see ‘Materials and Methods’ section).

**Figure 5.**
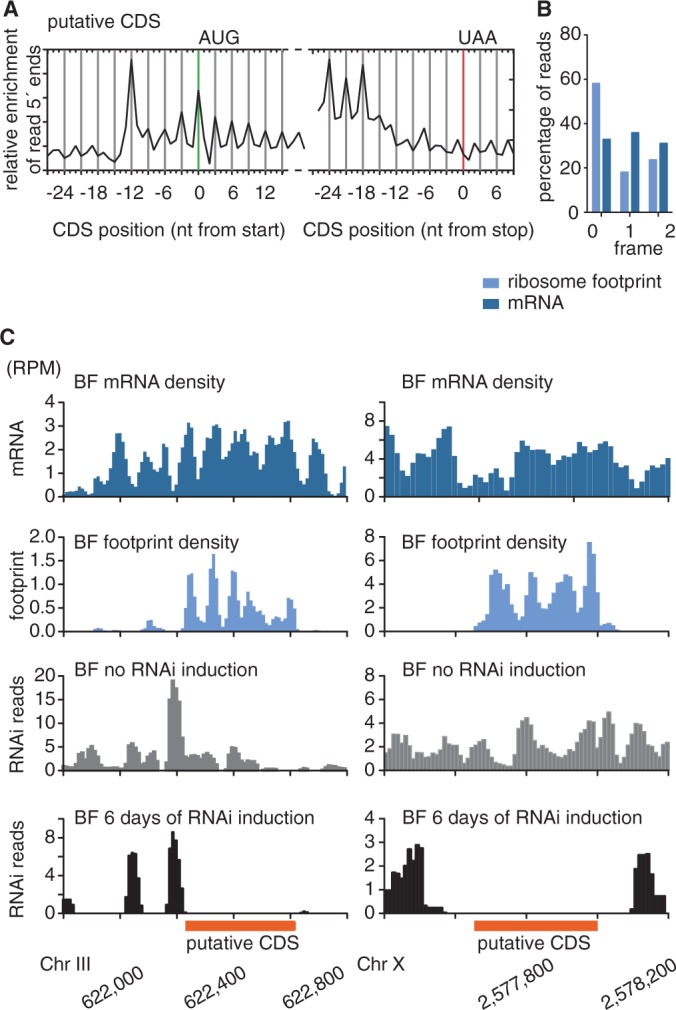
Ribosome footprints reveal previously un-annotated CDSs. (**A**) Alignment of the 5′ nucleotides from ribosome footprint reads that map close to translation start or translation termination sites of candidate CDSs. (**B**) Percentage of position of sequence reads relative to reading frame. (**C**) Ribosome, mRNA and RIT-seq (RNAi target sequencing) profiles of two previously un-annotated putative CDSs.

Similarly to annotated CDSs and uORFs, our averaged ribosome footprint data indicated a well-defined 3-nt periodicity for sequence reads occurring near translation initiation sites. However, we observed a less well-defined drop in ribosome densities across translation termination sites (Figure 5A and B), which may indicate a lack of productive translation. Because we observed an abrupt drop in footprint density downstream of the stop codon of annotated genes in *T. brucei* (Figure 1A and B), we decided to apply the same parameters to evaluate the likelihood that ORFs with high footprint density are indeed being translated into functional proteins. Again, candidate CDSs were defined as ORFs with a minimum length of 10 aa and an average ribosome footprint coverage of 2× over at least 70% of the ORF. To define the corresponding putative 3′ UTR, we applied the same parameters used in mice (53), i.e. the 3′ UTR was defined as the region beginning immediately downstream of the ORF and ending at the first subsequent start codon (in any reading frame). A limitation of the RRS is that it can only be calculated for an ORF if at least one entire footprint read and one RNA read map to the putative CDS and the corresponding 3′ UTR. Because many 3′ UTRs were too short to fulfill this criterion or simply did not contain any footprint reads, a sign for efficient translation termination, we were able to determine the RRS for only 1445 of genes annotated in *T. brucei* (Supplementary Table S7). For these 1445 genes the median RRS was 83.9 compared with an RRS of 0.19 for the subset of genes annotated as hypothetical unlikely (*N* = 90). Thus, similar to the observation in mice, the RRS appears to be a good predictor of productive translation. Next we determined the RRS for the previously un-annotated candidate CDSs that fulfilled the criteria for RRS calculation (*N* = 265). The median RRS of these candidate CDSs was with 3.99 lower than the median RRS of the annotated genes (83.9). Thirteen candidate CDSs had an RRS larger than 83.9 and 98 candidate CDSs had an RRS higher than the well-described intron-containing poly(A) polymerase (RRS = 9.63). The RRS could only be calculated for one of the candidate CDSs for which we had identified protein products (RRS = 57.44).

Finally, in an attempt to learn about the function of the putative CDSs, we searched for known protein motifs in those candidate CDSs for which we had identified protein products and for those with an RRS above 10. However, except for one candidate CDS located within the histone H2B gene array and which contained an H2B signature motif (Supplementary Table S5), no motifs were identified.

Taken together, proteomics data and a well-defined ribosome release (high RRS) suggest extensive translation beyond the annotated CDSs in *T. brucei*.

### Newly identified CDSs are important for parasite fitness

The combination of ribosome profiling data with previously published mass spectrometric data allowed us to identify a large number of new CDSs. Nonetheless, the biological significance of these CDSs remains to be determined. In addition, while ORFs with a high ribosome footprint density, a low RRS and without mass spectrometric evidence may not be translated into functional proteins, they may, nevertheless, play important regulatory roles.

Therefore, we decided to evaluate the importance of our candidate CDSs, independent of RRS and mass spectrometric evidence, in parasite survival. A previously published high-throughput phenotyping approach, termed RIT-seq, measured the fitness–cost associations using RNA interference (RNAi). The RIT-seq data revealed a significant loss in fitness on RNAi induction of 2724 CDSs in the BF, 1972 CDSs in the PF and 2677 CDSs in induced and differentiated parasites (54). The approach is based on the integration of RNAi libraries into trypanosomes and a comparison of the recovery of RNAi targets from trypanosome populations before and after RNAi induction. The RNAi library was generated using genomic DNA, but the effect on parasite fitness was only determined for annotated genes. Re-analysing the same datasets, we find a significant loss of fitness on RNAi induction for 214 putative CDSs in the BF (6 days after RNAi induction), 16 CDSs in the PF and 227 CDSs on differentiation (Supplementary Table S8). For examples of candidate CDSs potentially essential for viability in the BF see Figure 5C. It is important to note that the RNAi library used for the RIT-seq experiments contains a median insert size of 600 bp (average: 1 kb), thus somewhat limiting the resolution of the RIT-seq data (80,81). Nevertheless, these findings suggest an important biological role for a subset of genes that have been missed in previous genome annotations. Intriguingly, the RNAi data reveal large developmental differences in the fitness costs associated with the down-regulation of putative CDSs, suggesting that the set of candidate CDSs may be more important during the mammalian stage.

## DISCUSSION

In this study, we report the first genome-wide analysis of protein synthesis and a strand-specific analysis of RNA transcript levels for *T. brucei*. It is the first such analysis for a eukaryotic pathogen and an organism without transcriptional control. Our data reveal large differences in translational efficiency among transcripts in the same life cycle stage and between the same transcript in different stages. In addition, the sequencing of ribosome-protected RNA enabled us to identify transcripts that are likely to be translated. Hundreds of putative, previously unidentified CDSs appear to be essential for parasite fitness, and the analysis of available proteomics data confirmed the existence of at least 20 of these previously unknown CDSs.

Eukaryotic gene expression is regulated at multiple levels, with translation of mRNA into proteins being one of the most important (1,7). The ability to determine both translatome and transcriptome enabled us to evaluate the efficiency with which individual transcripts are translated into proteins and suggests translational control to be an important regulatory mechanism in *T. brucei*. Under a single condition, we observed translational efficiency to vary over two orders of magnitude; thus, its importance equals that of RNA stability for which a similar range has been measured (82). However, we observed no correlation between RNA abundance and translational efficiency, suggesting that translational efficiency is regulated independently of RNA stability.

To use ribosome density to estimate the rate of protein synthesis, the speed of translation must be constant. This assumption is supported by measurements in mouse embryonic stem cells that demonstrate the rate of translation to be consistent between different classes of mRNA, the kinetics of elongation to be independent of length and protein abundance and the speed of translation to be independent of codon usage (46,64). Nevertheless, the speed of translation may very well be different between the two life cycle stages of the parasite that live at 37°C and 27°C. Furthermore, our approach does not permit measurement of the absolute mRNA and footprint abundance for the individual life cycle stages, making direct comparisons of translation efficiencies for individual transcripts unreliable. Therefore, we did not compare translational efficiency directly, but rather determined the ‘rank’ in translational efficiency for all genes in each life cycle stage, assuming that a ‘change in rank’ indicated life cycle-dependent translational control. While we see a general positive correlation in translational efficiency between the two life cycle stages (Pearson r = 0.7428), for a large number of genes, we observed developmental regulation of translation. Importantly, the regulation we observe agrees well with that reported in previous studies for a small subset of genes but also includes many proteins previously unknown to be developmentally regulated. In addition, the efficient translation of proteins annotated as cytochrome oxidases in the PF and of enzymes important for glycolysis in the BF is consistent with differences in energy metabolism with the BF entirely dependent on glycolysis to generate energy.

How translational control is achieved in *T. brucei* remains to be seen, but our analysis suggests that uORF may be an important contributor. Work in several organisms has shown that generally the presence of uORFs correlates with reduced gene expression (71). However, for some genes, like the transcription factor GCN4 in yeast and the activating transcription factor ATF4 in mammals, this correlation is reversed on stress (83,84). Thus, uORF can exert positive and negative effects on translation of downstream CDSs. Based on previous 5′ UTR annotations, we identified uORFs in 22% of *T. brucei* genes, but it will be interesting to learn if the regulatory effects of uORFs may be supplemented by translation from non-AUG uORFs. Our ribosome profiling data indicate an increase in translation from 5′ UTRs and a more ubiquitous translation initiation from non-AUG sites in the PF compared with the BF. Similarly, an increase in the translation from 5′ UTRs has been observed in yeast on starvation (45). One of the most highly conserved stress-induced mechanisms to regulate translation involves the inactivation of the translation initiation factor eIF2α by phosphorylation at a conserved serine (85,86). Phosphorylation of the *T. brucei* and *L. major* orthologs occurs at the corresponding Thr residues (6,87), but the generation of a *T. brucei* cell line exclusively expressing a mutant form of eIF2α demonstrated that phosphorylation of eIF2α is not required for heat-induced translational arrest or for the formation of heat shock stress granules in *T. brucei* (88). Given the finding that eIF2α may also be important in determining the stringency in initiator codon selection (89), it will be interesting to see whether eIF2α phosphorylation affects the selection of initiator codons and whether this plays a role in the fine-tuning of gene expression.

Besides being a valuable tool to study translational regulation, ribosome profiling will also be invaluable for the discovery of small peptides. While only ∼2% of the human genome contains annotated CDSs (90), transcriptome analyses have revealed almost genome-wide transcription of RNA, mostly considered to be non-protein-coding. In lower organisms the percentage of the genome encoding for proteins is higher (62,91,92), but pervasive transcription of non-protein coding regions can be found in essentially all organisms (93). The vast amount of ncRNA has triggered great interest in elucidating the biological significance of these transcripts and has led to the identification of numerous regulatory mechanisms controlled by short and long ncRNA. Interestingly, the majority of transcription occurs as long ncRNA in humans (94), many of which contain ORFs making it difficult to unambiguously classify a transcript as non-protein-coding. To distinguish long ncRNA from mRNA, ORF length has served as the most commonly applied criterion, because even without selective pressure, short ORFs will occur frequently by chance while the probability that long ORFs occur by chance are low. Nevertheless, recent findings reveal that eukaryotic genomes contain a large number of small CDSs (95–97) and that small peptides play important biological roles (77,78,98,99). In addition, numerous long ncRNAs containing ORFs longer than 100 aa have been found to associate with ribosomes (46,53). While these findings complicate the annotation of CDSs and demand for new approaches to annotate genes, they raise the exciting possibility that a large number of unknown small CDSs exists and awaits discovery.

For this study, RNA was obtained from a 427 strain, the most common laboratory strain, and sequence reads were aligned to the more complete genome of the 927 strain; nevertheless, RNA-sequencing analysis indicates widespread transcription. Thus, as seen in other organisms, active transcription is not necessarily a good predictor of CDSs. In contrast, we found ribosome footprint density to be highly enriched across annotated CDSs, excluding introns. It should thus serve as a reliable tool to identify novel genes. To search for new CDSs and to evaluate their biological function, we combined ribosome profiling, proteomics and genome-wide RNAi data. While proteomics data allowed us to identify protein products for only 20 relatively large candidate CDSs, the experimental set-up of the proteomic analysis that involved the removal of small peptides made it impossible to verify the existence of many small CDSs. Thus, more important than the verification of putative CDSs with proteomics data may be our finding that >200 of the putative CDSs appear to be essential for parasite fitness. Furthermore, RIT-seq data suggest that candidate CDSs are 13-fold less likely to be essential for viability in the PF than in the BF or during differentiation of cells from BF to PF. These findings point to a possible role of small peptides in the adaptation of trypanosomes to life in the mammalian host. Generally, our data suggest the existence of a large yet to be explored small proteome, but further characterizations of the small proteome will have to include a more targeted proteomics analysis.

In this analysis, we focused on measuring translational control and the identification of new CDSs. However, ribosome profiling data should also aid in the identification of *bona fide* ncRNAs. For many annotated CDSs we observed RNA transcripts but not ribosome footprints, suggesting the presence of ncRNA. Though, given that we have only analysed two life cycle stages, these putative ncRNAs may be translated during other stages of the parasite’s life cycle. Thus, a comprehensive search for ncRNA should include ribosome profiling analyses of all life cycle stages.

## ACCESSION NUMBERS

All sequencing data have been deposited in the European Nucleotide Archive: PRJEB4801.

## SUPPLEMENTARY DATA

Supplementary Data are available at NAR Online.

## FUNDING

Young Investigator Program of the Research Center of Infectious Diseases (ZINF) of the University of Wuerzburg, Germany; German Research Foundation DFG [SI 1610/2-1]; Human Frontier Science Program (to T.N.S.); French National Research Agency [ANR-2010-GENM-011-01, GENAMIBE to C.C.H.]. Funding for open access charge: German Research Foundation (DFG) and the University of Wuerzburg in the funding programme Open Access Publishing.

*Conflict of interest statement*. None declared.

## Supplementary Material

Supplementary Data
