# Supplementary material for: Comparative ribosome profiling reveals extensive translational complexity in different Trypanosoma brucei life cycle stages
Source: Nucleic Acids Res. 2014 Jan 17;42(6):3623–37. doi: 10.1093/nar/gkt1386 (PMC3973304; doi:10.1093/nar/gkt1386)
Supplement: Supplementary Data [file supp_42_6_3623__index.html]

Comparative ribosome profiling reveals extensive translational complexity in different Trypanosoma brucei life cycle stages — Supplementary Data 

# Comparative ribosome profiling reveals extensive translational complexity in different *Trypanosoma brucei* life cycle stages

## Supplementary Data

files

**Files in this Data Supplement:**

- Supplementary Data - pdf file
- Supplementary Data - xlsx file
- Supplementary Data - xlsx file
- Supplementary Data - xlsx file
- Supplementary Data - xlsx file
- Supplementary Data - xlsx file
- Supplementary Data - xlsx file
- Supplementary Data - xlsx file
- Supplementary Data - xlsx file
