# Supplementary material for: Comparative ribosome profiling reveals extensive translational complexity in different Trypanosoma brucei life cycle stages
Source: Nucleic Acids Res. 2014 Jan 17;42(6):3623–37. doi: 10.1093/nar/gkt1386 (PMC3973304; doi:10.1093/nar/gkt1386)

SUPPLEMENTARY TABLE AND FIGURE LEGENDS

Figure S1. Sucrose density gradient analysis of undigested and nuclease-digested extracts from PF and BF.

Figure S2. Establishment of a ribo-seq protocol. (A) Outline of the protocol used to convert ribosome footprints and fragmented mRNA into sequencing libraries. Adapted from Ingolia et al. 2009. (B) Pair-wise comparison of ribosome footprint density from BF (upper panel) and PF (lower panel) cell extracts digested at RT and 4° C. Density is measured as reads per kilobase per million reads (rpkm) to normalize for differences in gene length and library size. The two genes marked in red are hypothetical genes that overlap with rRNA genes.

Figure S3. Meta-gene analysis of ribosome density. Alignment of the 5´ nucleotide from ribosome footprint reads that map close to translation start or translation termination sites. To calculate average scaled count of read 5´ ends, the peak count within each gene was scaled to 1. The scaled counts at each position of all genes were summed up and then divided be the number of genes.

Table S1
mRNA levels and ribosome footprint levels across annotated CDSs. mRNA levels are listed as read counts per gene (sheet 1), reads per million reads per kb (sheet 2) and transcripts per cell (sheet 3). Ribosome footprint levels are listed as footprint read counts per gene (sheet 1), footprint read counts per million reads per kb (sheet 2) and ribosomes per gene per cell (sheet 3).

Table S2
Translational efficiency for annotated CDSs. RNA levels and ribosome levels are listed as reads per million reads per kb.

Table S3
Life-cycle specific rank in translational efficiency.

Table S4
RNA-seq, ribo-seq and proteomics data for uORF.

Table S5
RNA-seq, ribo-seq and proteomics data for previously unannotated putative CDSs.

Table S6
mRNA levels and ribosome footprint levels across transcripts identified by Kolev et al. 2010, PloS Pathogen.

Table S7
Ribosome release score for annotated CDSs, uORF and previously unannotated putative CDSs.

Table S8
Fitness-associated costs of RNAi against previously unannotated putative CDSs in PF, BF and differentiated parasites.

Supplementary Figure S1

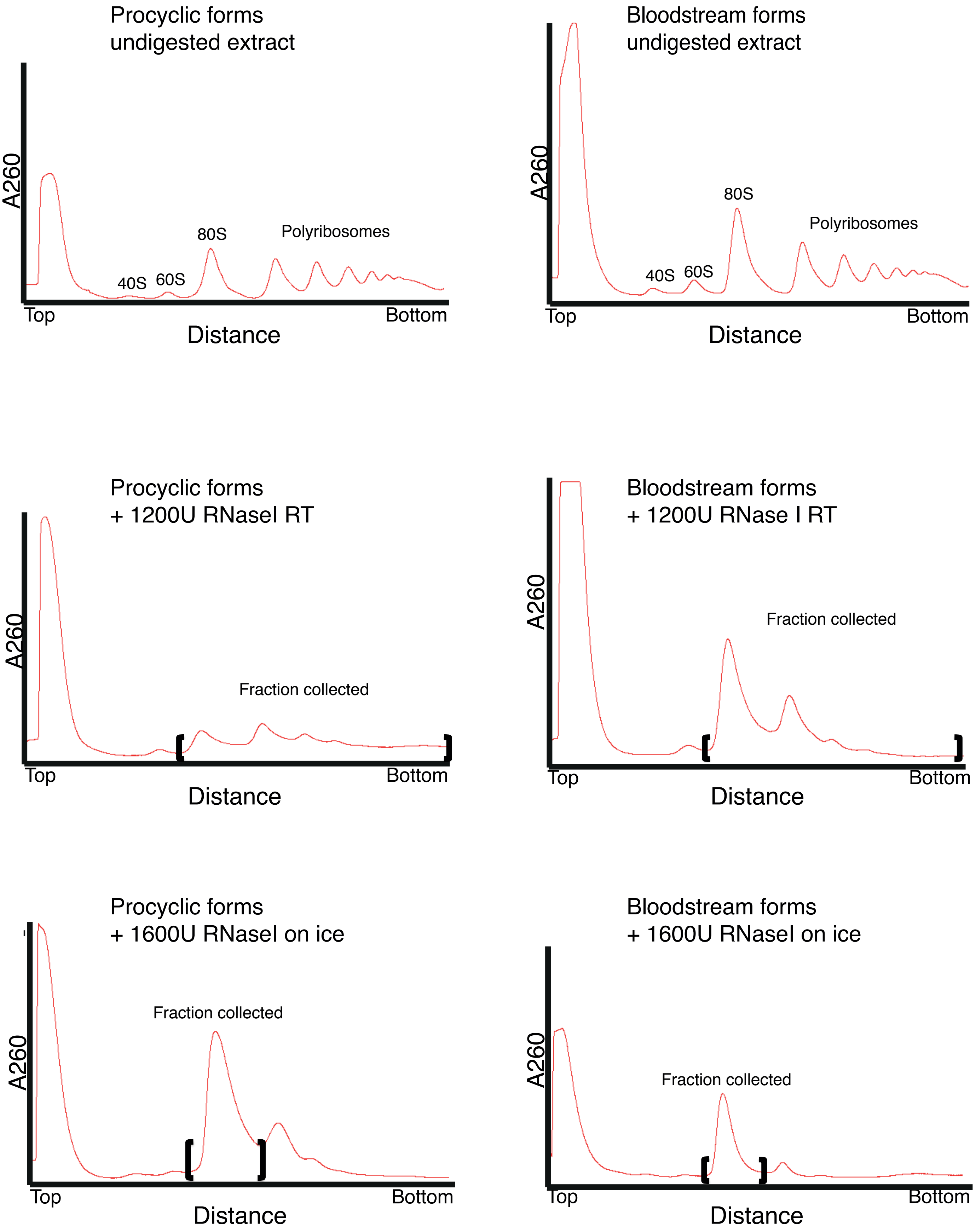

Supplementary Figure S2

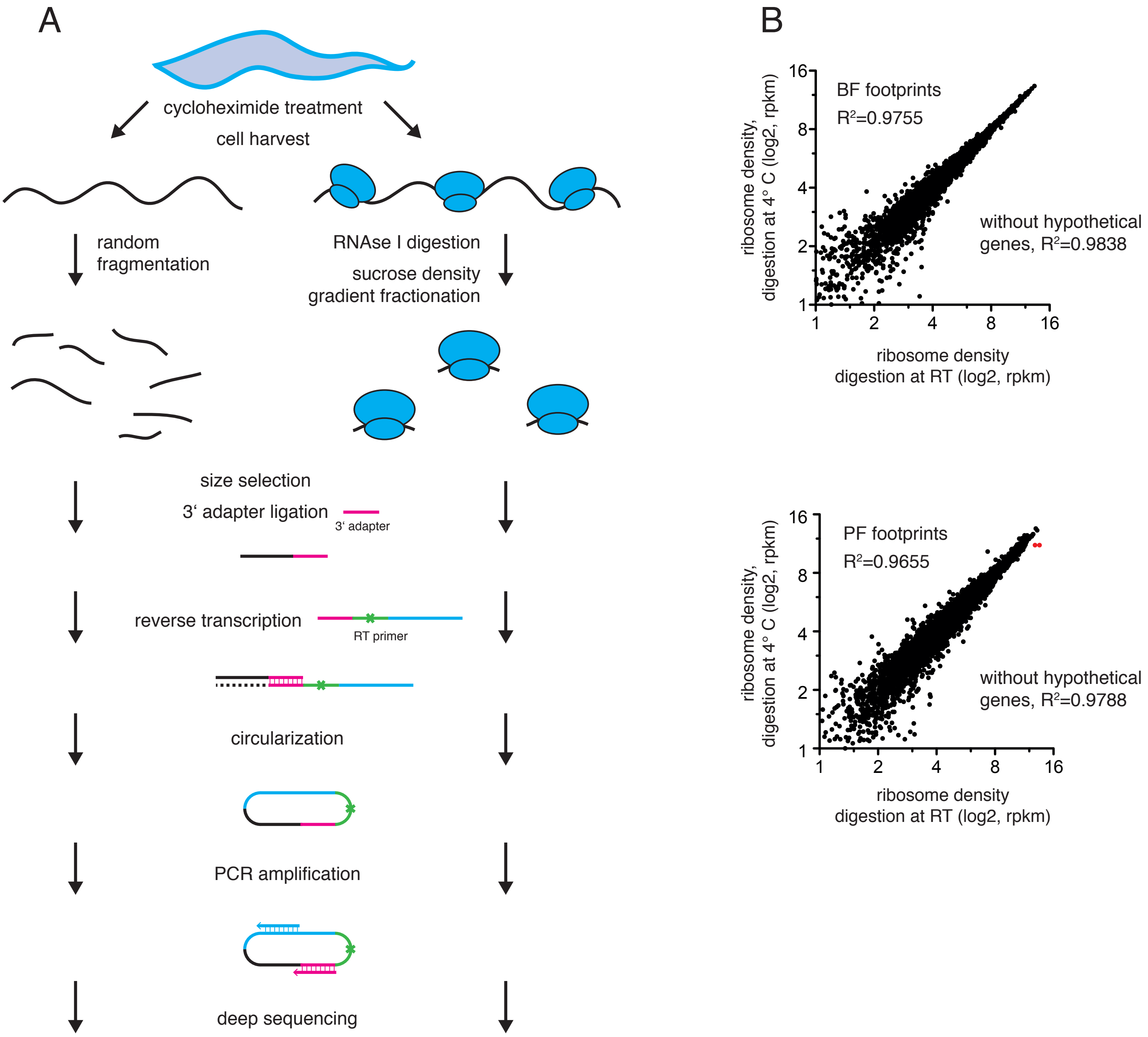

Supplementary Figure S3

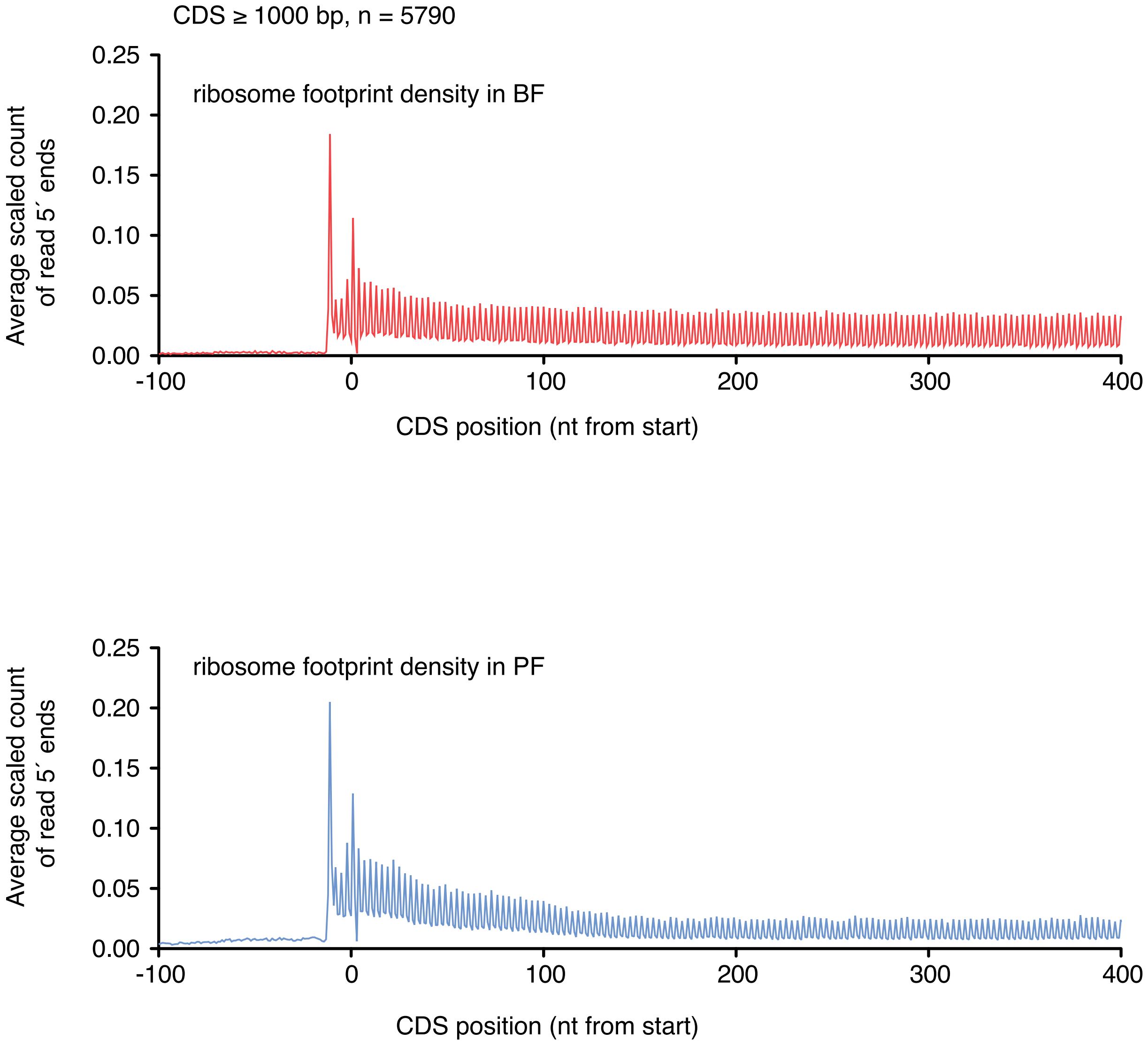

Supplement: Supplementary Data [file supp_gkt1386_Supplementary_Legends_Figures.pdf]
